# Atranorin Fluorescence: A Possible Photoprotective Role in Lichen Defense Against UV and Blue–Violet Radiation

**DOI:** 10.3390/jof12100768

**Published:** 2026-10-13

**Authors:** Ilya A. Prokopiev, Gregory A. Pozhvanov, Alexandra N. Ivanova, Zhanna O. Zholobova, Daria A. Frolova, Alexey L. Shavarda

**Affiliations:** 1 Laboratory of Analytical Phytochemistry, Komarov Botanical Institute of the Russian Academy of Sciences, 197022 St. Petersburg, Russia; zzholobova@binran.ru (Z.O.Z.); shavarda@binran.ru (A.L.S.); 2 Department of Biological and Medicinal Chemistry, Institute of Experimental Medicine, 197376 St. Petersburg, Russia; 3 Department of Botany and Ecology, Faculty of Biology, Herzen State Pedagogical University, 191186 St. Petersburg, Russia; 4 Laboratory of Anatomy and Morphology, Komarov Botanical Institute of the Russian Academy of Sciences, 197022 St. Petersburg, Russia; aivanova@binran.ru; 5 Center for Molecular and Cell Technologies, Research Park, St. Petersburg State University, 199034 St. Petersburg, Russia; 6 Chemical Analysis and Materials Research Centre, Research Park, St. Petersburg State University, 199034 St. Petersburg, Russia; d.a.frolova@spbu.ru

**Keywords:** *Hypogymnia*, secondary metabolites, atranorin, chloroatranorin, light reflection, fluorescence, photoprotection, chlorophyll fluorescence

## Abstract

Epiphytic lichens are naturally subjected to highly fluctuating light and must protect their algal symbionts from harmful short wavelengths while facilitating efficient photosynthesis. Confocal microscopy confirmed that the upper cortices of *Hypogymnia physodes* and *H. bitteri* thalli contained colorless secondary metabolites atranorin and chloroatranorin, which were able to protect lichens against excessive UV and blue–violet radiation in two ways. The first way screens algae through visible light reflection; the second way is realized through the absorption of UV-A and blue–violet radiation and fluorescence emission in the yellow–green spectrum. Acetone rinsing removed atranorin and chloroatranorin crystals from the cortical surface, thus reducing the reflectance of both *H. physodes* and *H. bitteri*. Nevertheless, fluorescence in *H. physodes* remained largely unaffected due to the deep-layer localization of atranorin within the upper cortex. However, melanized *H. bitteri* exhibited weak fluorescence either before or after acetone rinsing, despite containing atranorin and chloroatranorin at levels comparable to *H. physodes*. Within the heavily melanized lower cortical layer of *H. bitteri*, young areas contained significant amounts of atranorin and chloroatranorin. A reduction in their content resulted in a compensatory enhancement of non-photochemical quenching in algae. This phenomenon suggests that atranorin and chloroatranorin contribute to the photoprotection of lichens in yet another way through light reflectance and through fluorescence in a photosynthetically safe manner.

## 1. Introduction

Lichens are producers of a large number of specific secondary metabolites, the majority of which are not found in other living organisms [1,2]. Currently, more than 1000 secondary low-molecular-weight metabolites of lichens are known [2,3]. There are three main biosynthesis pathways of secondary lichen metabolites: acetyl-polymalonyl, mevalonic acid, and shikimic acid [4]. Most secondary metabolites of lichens (about 500 compounds) belong to derivatives of the acetyl-polymalonyl pathway [2].

It is known that aromatic secondary metabolites of lichens are capable of absorbing radiation in the UV-B (280–315 nm) and UV-A (315–400 nm) ranges, which reach the Earth’s surface [5]. It is assumed that UV absorption is one of the main protective functions of secondary lichen metabolites that accumulate in the upper cortex [6]. However, photodamage is induced not only by UV irradiation but also by excessive visible light exposure, particularly in the blue–violet spectrum [7,8]. It is known that some colored lichen pigments, such as usnic acid, parietin, vulpinic acid and melanins, are able to absorb—in addition to UV radiation—a part of the visible blue–violet wavelengths [9,10,11]. Furthermore, it has been shown that the widespread colorless β-orcinol depside atranorin is capable of screening throughout the visible spectrum via light reflection [10,12]. Authors hypothesized this to be a result of Mie scattering from atranorin crystals at the hyphal interfaces [10]. Secondary aromatic lichen metabolites can absorb UV-A radiation excesses, and evince the property of emitting visible fluorescence [13]. Fluorescence under UV-A has proved to be a valuable tool in the routine examination of lichen substances: xanthones emit fluorescence in shades of yellow, orange and red, while depsides and depsidones generally emit in blue-to-white or in shades of gray, although atranorin shows a yellow hue [14]. Also, atranorin was presumed to convert UV radiation into photosynthetically usable light for the photobiont, showing maximum fluorescence at 425 nm under 400 nm excitation in toluene [15]. Recent studies demonstrate that parietin, an anthraquinone localized in the upper cortex of *Xanthoria parietina*, absorbs UV-B radiation (300 nm) and exhibits maximum fluorescence emission in the orange spectrum at 610 nm [16]. However, the role of parietin in the conversion of UV radiation energy into photosynthetically active wavelengths is not supported. Thus, many secondary lichen compounds are fluorescent, but the physiological effect of this fluorescence in lichens remains unknown.

The present work aims to investigate the photoprotective role of secondary metabolites fluorescence of lichens *Hypogymnia physodes* and *H. bitteri* against UV-A and blue–violet radiation.

## 2. Materials and Methods

### 2.1. Lichens Collection

Specimens of *Hypogymnia physodes* (L.) Nyl. (1896) were collected in Northern Karelia at the White Sea Biological Station “Kartesh” (66.33667° N, 33.64583° E), on branches of *Pinus sylvestris* L. (HP1), and at the right bank of the Eastern Khandyga River (63.09634° N, 137.75648° E) on bark of *Duschekia fruticosa* (Rupr.) Pouzar (HP2). Specimens of *Hypogymnia bitteri* (Lynge) Ahti were collected in East Yakutia at the right bank of the Eastern Khandyga River on bark of *Larix gmelinii* (Rupr.) Rupr. (HB1) and *Duschekia fruticosa* (HB2) at coordinates 63.13474° N, 138.78458° E and 63.14419° N, 158.53847° E, respectively. At least five specimens of each *Hypogymnia* species were sampled from each location.

### 2.2. Acetone Rinsing

Acetone rinsing was conducted according to the method described by Solhaug et al. [17]. Intact lichens were initially placed in a desiccator containing dry waterless calcium chloride for 24 h to ensure complete desiccation. Then, dry thalli were longitudinally dissected into two equal parts, one of which served as the control sample, and the other one was subjected to acetone rinsing. In all experiments, each air-dried thallus was rinsed four times with 30 mL of acetone, with each rinse lasting 5 min. After acetone rinsing, the treated lichens were left at room temperature for 12 h in darkness to allow residual acetone to evaporate before subsequent experiments.

### 2.3. Reflectance Spectrum Measurements

Reflectance spectra (420–730 nm) were recorded directly on the upper surface of each thallus, both acetone-rinsed and untreated, using an X-Rite ColorMunki spectrophotometer (X-Rite Inc., Grand Rapids, MI, USA) in reflective high-resolution mode under the control of spotread console utility from ArgyllCMS software package (v. 2.0.1 for macOS 10.14.6). Measurements were taken at five randomly selected positions of each thallus. Lichen samples were placed on a black fabric background to minimize stray light interference, and the distance between the upper thallus surface and the spectrometer detector was maintained at 0.5 cm. The spectrophotometer was calibrated using its internal reflective standard automatically prior to each measurement.

### 2.4. Fluorescence Spectrum Measurements

Lichen thalli or pure isolated secondary metabolite fluorescence spectra were recorded in darkness under excitation by Convoy C8 UV-A LED flashlight (330–415 nm, with the maximum at 365 nm; Hongkong Convoy Elextronics Co. Limited, Hong Kong, China). UV-A LED was mounted at a constant 14 cm distance from the sample surface at 20° angle. Fluorescence emission spectra were recorded directly from the upper thallus surface using the same X-Rite ColorMunki spectrophotometer in emissive high-resolution mode under the control of spotread console utility (see above). The distance between the sample surface and the spectrometer detector was maintained at 1 cm. To measure the fluorescence spectrum of individual lichen substances from the Komarov Botanical Institute pure secondary lichen substance collection, 10 mg of the substance was placed on a microscopy glass slide (Menzel/ThermoFisher Scientific GmbH, Dreieich, Germany) and distributed equally to form a spot 0.5 mm in thickness and at least 1 cm in diameter to fully correspond the device’s measurement port diameter (5 mm). Blank emission spectra of UV-illuminated black cloth background (for lichen thalli) or empty slide glass (for pure substances) were subtracted from the corresponding spectra prior to analysis. Vittatolic acid, 2′-O-methylphysodic acid, and α-alectoronic acid were not studied due to their insufficient quantities in the available collection of lichen substance standards.

### 2.5. PAM Fluorimetry

Photosynthetic capabilities of lichen thalli were characterized by pulse-amplitude modulation (PAM) fluorimetry using a Walz Junior-PAM portable device (Heinz Walz GmbH, Effeltrich, Germany) controlled by WinControl 3.35 software. After dark adaptation for 30 min, young peripheral areas of lichen thalli at 1–2 mm distance from the thallus edge were selected for illumination with a light guide (0.4 m length, 1 mm diam). PAM fluorometer was operated in “Light Curve” mode, in which the intensity of actinic light programmatically increased from zero and then from 66 μM photons·m^−2^·s^−1^ (low light) to 125 μM photons·m^−2^·s^−1^ (low–moderate light) to 285 μM photons·m^−2^·s^−1^ (moderate light) in 10 s steps. Photosystem II quantum yield (Y(II)), electron transfer rate (ETR) through electron transport chain of chloroplast, photochemical quenching (qP) and non-photochemical quenching (NPQ) were derived for further analysis [18].

### 2.6. Lichen Sample Preparation and Secondary Metabolite Extraction

For the analysis, air-dried acetone-rinsed thalli and intact control ones were divided into 5 equal parts, placed in Eppendorf 2.0 mL microcentrifuge tubes, then thoroughly ground in a M-0.05 vibration ball mill with two 3 mm steel balls per tube (Techno-Center LLC, Rybinsk, Russia). A total of 10 mg of the resulting powder were extracted twice with 1 mL of acetone. The extraction was carried out with constant stirring for 24 h at a temperature of 20–25 °C in an RM-1L vortex (ELMI SIA, Riga, Latvia) in the “F5” cycle mode (135° clockwise rotation, high-amplitude shake for 1.5 s, 90° clockwise rotation, high-amplitude shake for 1.5 s, 135° clockwise rotation, 1.5 s rest; 10 RPM in total). The obtained extracts were centrifuged at 15,000× *g* for 5 min and then the supernatant was kept at 4 °C prior to analysis.

### 2.7. HPLC-ESI-MS Analysis

High-performance liquid chromatography–electrospray ionization–mass spectrometry (HPLC–ESI–MS/MS) analysis was performed with a Shimadzu LC-30 Nexera chromatograph (Shimadzu Corporation, Kyoto, Japan). For chromatographic separation, a Phenomenex Kinetex C18 column (100 × 2.1 mm, 1.7 µm) was used (Phenomenex Inc., Torrance, CA, USA. The mobile phase consisted of (A) LCMS-grade water with 0.1% (*v*/*v*) formic acid and (B) LCMS-grade acetonitrile with 0.1 (*v*/*v*) formic acid. Analyses were performed at 40 °C with a flow rate of 0.3 mL/min in the gradient elution mode; the percentage of B was programmed as follows: 30% (2 min)–80% (15 min)–100% (16 min)–100% (20 min)–30% (20.1 min)–30% (25 min, controller stop). The volume of injected sample was 2 µL. Spectra of eluting substances were recorded in UV at 254 nm. After chromatographic separation, the samples were analyzed using a triple quadrupole mass-selective detector Shimadzu LCMS-8030 with electrospray ionization (ESI) in the negative mode. The voltage on the capillary was 3.5 kV, nebulizing gas flow rate 3 L/min, drying gas (nitrogen) temperature 250 °C, drying gas flow rate 15 L/min. Mass spectra were recorded in the range 100–800 *m*/*z*. MS/MS spectra were obtained by collision-induced dissociation of precursor ions with an energy of 30 eV. The resulting chromatograms were processed using the MZmine 4 v. 4.1.0 software. To identify lichen substances, we compared of their polarity related to their retention time (Rt), obtained *m*/*z* values of pseudo-molecular and fragment ions with the authentic standards from the Komarov Botanical Institute pure secondary lichen substances collection.

### 2.8. Confocal Microscopy

Fluorescent secondary substance distribution within lichen thalli was visualized with confocal laser scanning fluorescent microscopy. Lichens were moistened in Petri dishes between two layers of wet filter paper for 2 h, then thalli peripheral (young) part 5 mm fragments were embedded into 3% (*w*/*v*) agarose gel cooled down to +40 °C. Small cubical fragments of agarose gel with embedded lichen thalli were transversally sectioned into 30 μm slices using Leica VT1200S vibratome (Leica Microsystems, Wetzlar, Germany). Lichen sections were positioned on microscope slides, filled with TRIS/MES buffer (0.4 mM/0.2 mM, pH 6.0), covered with coverslips and stored in darkness at +6 °C for examination within 12 h. Lichen thalli sections were examined using a Carl Zeiss LSM 780 laser scanning confocal microscope (Carl Zeiss Microscopy GmbH, Jena, Germany) under the Plan-Apochromat 20× objective lens (NA 0.8). Atranorin and chlorophyll fluorescence was induced with a 405 nm diode laser, and their emission was recorded in ranges of 525–595 nm and 675–705 nm, respectively. The monochrome background image was rendered by 594 nm helium–neon laser transmitted light. Laser lines were mitigated with MBS-405 and MBS-488/594 beam splitter filters. Z-stacks of 10 to 18 optical sections (303.64 × 303.64 μm) were recorded at 2.5 μm intervals. Figures show maximal intensity projections of a substack from the central part of the acquired z-stack, if not indicated otherwise. Confocal image morphometry was done in Fiji v. 2.16.0 (ImageJ 1.54t; NIH, Bethesda, MD, USA) using the Straight Line tool. Data acquisition, substacking and maximal projection preparation were performed in the manufacturer’s software (ZEN Black v. 3.8 2023, Carl Zeiss Microscopy GmbH), and final images were assembled in ZEN Blue software (v. 3.8 2023) by overlaying the maximal intensity projection of fluorescent channels over a single most sharp monochrome transmissive image.

### 2.9. Statistics

The results of all experiments are presented as arithmetic means and standard deviations (M ± SD). The statistical significance of differences in the mean values was determined by non-parametric Mann–Whitney tests using the Real Statistics resource pack for Microsoft Excel (version 16.78). Statistical significance was assessed at α = 0.05.

## 3. Results

### 3.1. Content of Secondary Metabolites in the Studied Lichens

**Figure 1:**
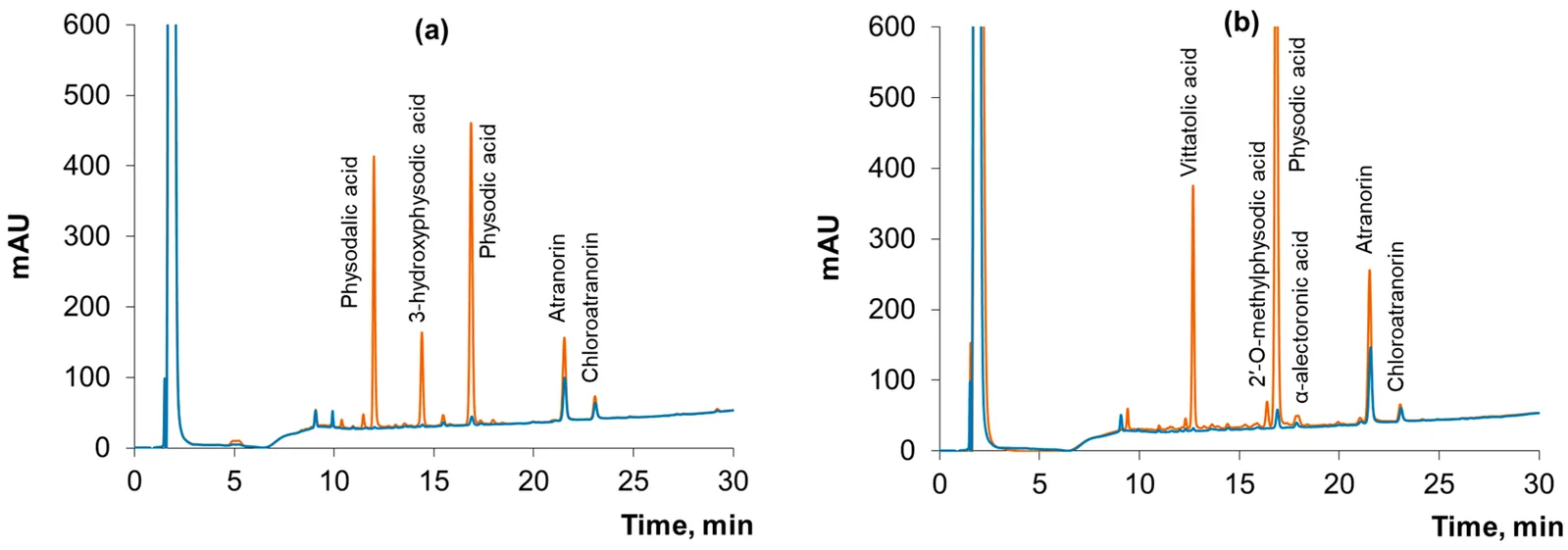
HPLC chromatograms (250 nm) of acetone extracts of lichens *Hypogymnia physodes* (HP2) (**a**) and *H. bitteri* (HB1) (**b**), before (orange line) and after (blue line) acetone rinsing.

**Figure 2:**
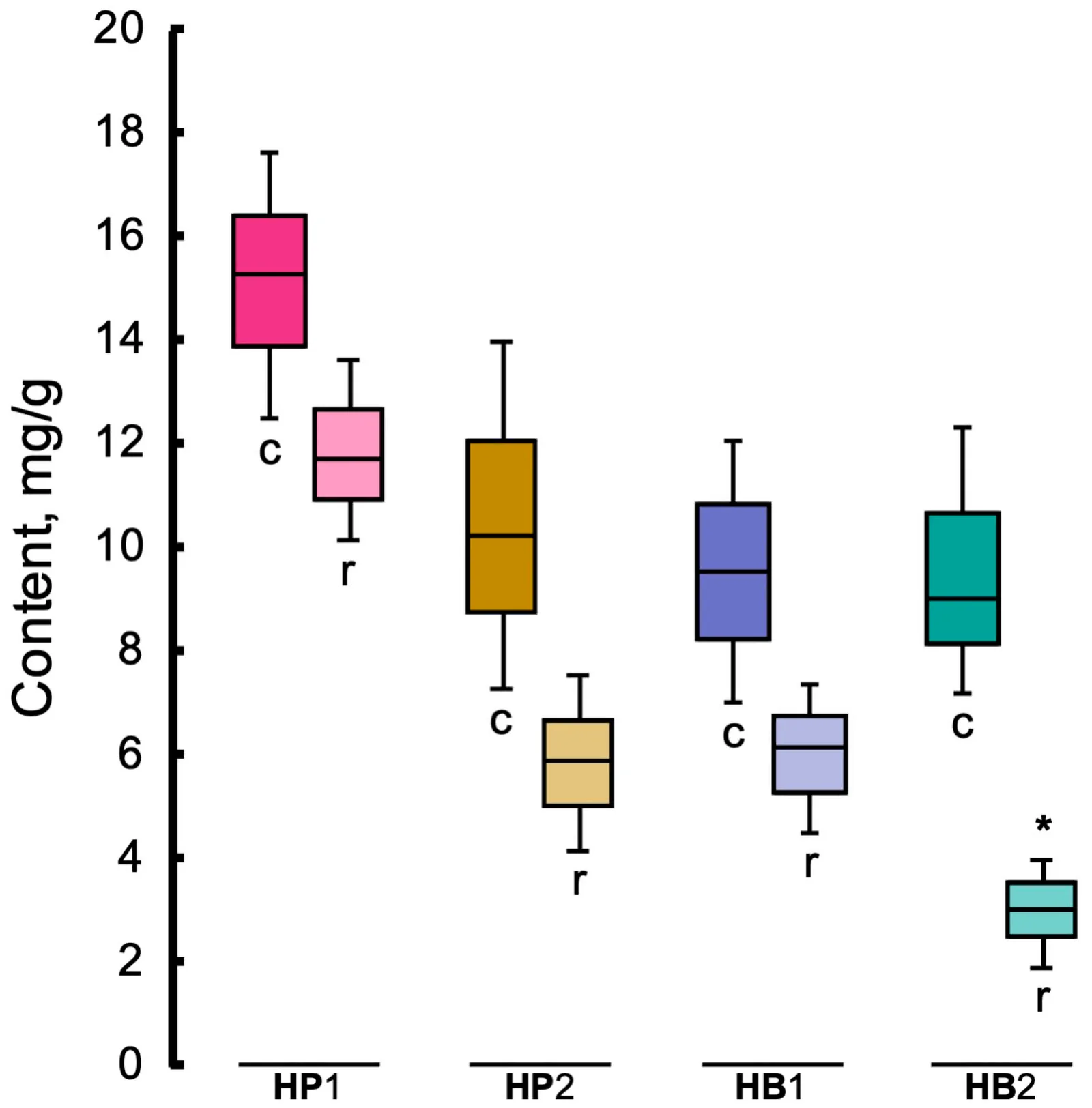
Total content of atranorin and chloroatranorin in the air-dried samples of *Hypogymnia physodes* (HP1 and HP2) and *H. bitteri* (HB1 and HB2) before (control, marked with “c” letter) and after acetone rinsing (marked with “r” letter). Asterisk marks the groups whose values are significantly different from the corresponding control (*n* = 5, *p* < 0.05, Mann–Whitney test).

Eight secondary metabolites were identified in the studied specimens of *Hypogymnia physodes* and *H. bitteri*: physodalic acid, vittatolic acid, 3-hydroxyphysodic acid, 2′-O-methylphysodic acid, physodic acid, α-alectoronic acid, atranorin and chloroatranorin (Table 1). Chromatographic analysis revealed that acetone rinsing of thalli of the studied lichen species resulted in near-complete (90–99% based on a comparison of peak areas) removal of depsidones accumulating in the medullary layer (Figure 1). In contrast, atranorin and chloroatranorin, localized in the cortical layer, were less effectively removed by acetone (Figure 1). Quantitative assessment of untreated thalli demonstrated that the total content of atranorin and chloroatranorin varied within a broad range of 6–18 mg/g dry weight. Acetone rinsing reduced atranorin and chloroatranorin total content by 1.2–2.2 times in *Hypogymnia physodes* and 1.5–3.0 times in *H. bitteri* compared to untreated controls (Figure 2).

### 3.2. Reflectance Spectra of the Studied Lichens Before and After Acetone Rinsing

**Figure 3:**
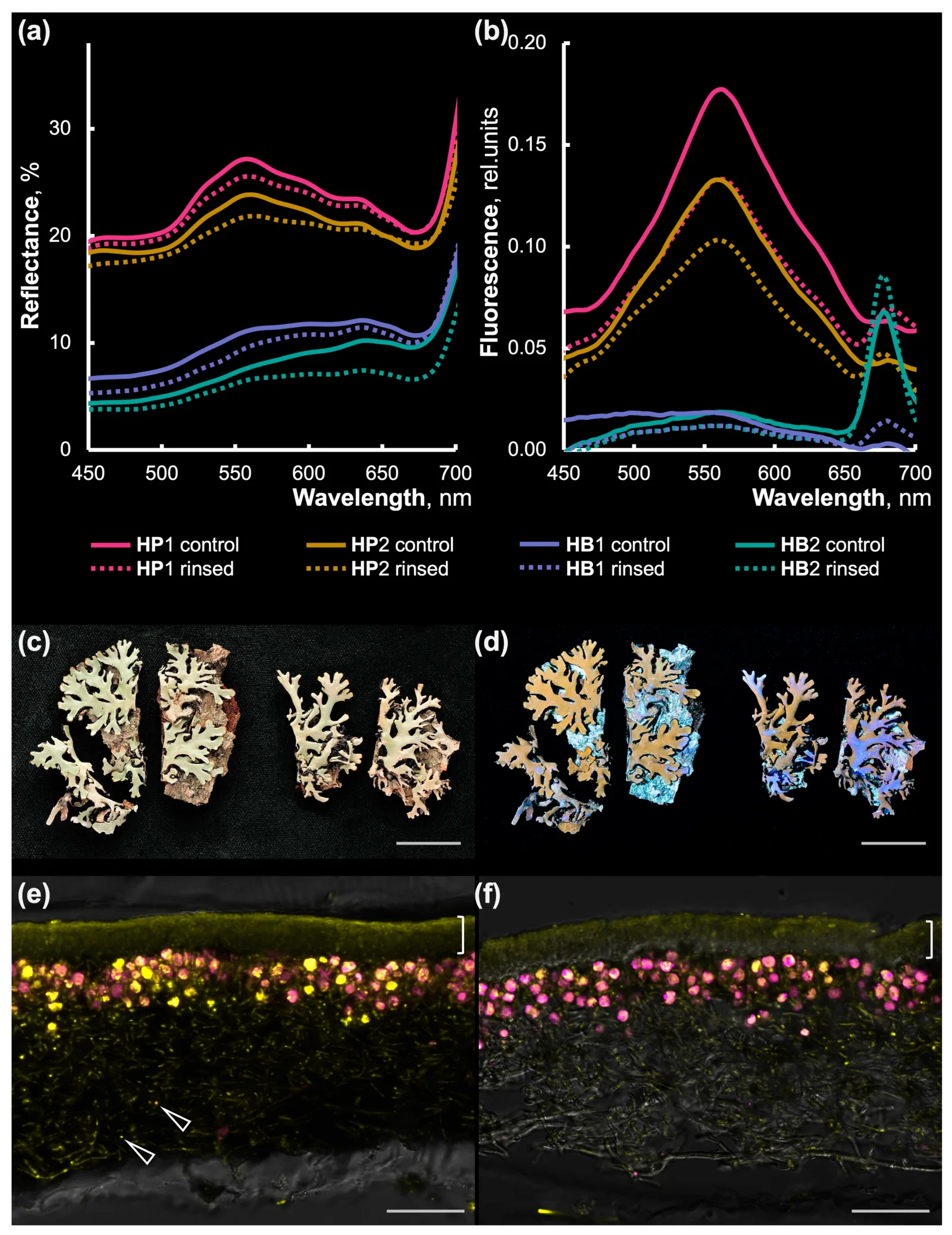
Spectral reflectance and fluorescence curves of *Hypogymnia physodes* and *H. bitteri* thalli surfaces, their appearance under visible light and UV, and atranorin and chloroatranorin localization in thalli. (**a**) Spectral reflectance and (**b**) fluorescence curves of the air-dried upper thallus surfaces in *Hypogymnia physodes* (HP1 and HP2) and *H. bitteri* (HB1 and HB2) before (control) and after acetone rinsing. Each curve in (**a**,**b**) represents the mean of 5 replicate measurements. (**c**,**d**) Appearance of HP1 air-dried (**left**) and acetone-rinsed (**right**) thalli under visible light (**c**) and UV (365 nm) (**d**). (**e**,**f**) Atranorin and chloroatranorin localization in cross-section of control (**e**) and acetone-rinsed (**f**) HP1 thalli. Images represent maximal intensity projections from 8 to 14 confocal optical sections. Yellow channel: atranorin and chloroatranorin fluorescence excited by 405 nm diode laser; magenta channel: chlorophyll autofluorescence excited by the same laser. Monochrome background: transmission channel rendered by 594 nm laser. Arrows indicate fluorescent secondary metabolites accumulated in spots in medullary layer. Brackets indicate thallus upper cortex. Scale bars are 1 cm in (**c**,**d**) and 50 μm (**e**,**f**).

Acetone rinsing of *H. physodes* and *H. bitteri* thalli resulted in a 5–20% reduction (on average) in thallus surface reflectance across all studied samples compared to the acetone-untreated control (Figure 3a). Lichens with gray-colored thalli, *H. physodes* (HP1 and HP2), demonstrated, on average, twice the reflectance of incident radiation compared to partially melanized *H. bitteri* (HB1 and HB2), both before and after acetone rinsing (Figure 3a,c). *H. physodes* thallus reflectance spectra exhibited the local maximum at 560 nm in the visible range, and a local minimum within 670–680 nm (Figure 3a). Similarly, *H. bitteri* had the same local minimum in the red part of visible range; however, their reflectance curve did not have any pronounced peaks in the visible range. Both species showed increasing reflectance near the infrared range of the spectrum, similarly to the reflectance properties of higher plant leaves.

### 3.3. Fluorescence Spectra of the Lichens Before and After Acetone Rinsing

Illumination of *H. physodes* and *H. bitteri* thallus surface by UV-A and blue–violet wavelengths (330–415 nm) induced fluorescence, which was especially well-visible in *H. physodes* (Figure 3d). In the fluorescence spectra, an emission maximum at 563 nm in the green–yellow range of the visible spectrum was revealed for both *Hypogymnia* species (Figure 3b). Acetone rinsing of the *H. physodes* thalli led to a 1.3- to 1.4-fold decrease in fluorescence compared to the untreated control. However, in the case of *H. bitteri*, fluorescence was either weak (HB1) or not observed (HB2) both before and after acetone rinsing. Importantly, the long-wavelength region of *H. physodes* and *H. bitteri* fluorescence spectra also revealed a signal increase at 680 nm, the area to which the fluorescence peak of P680 chlorophyll belongs to (Figure 3b). Among intact thalli, this 680 nm peak was minor in HP1, HP2 and HB1, but in HB2 this peak was well-pronounced, corresponding to the lowest detected content of atranorin and chloroatranorin (Figure 2). Remarkably, acetone rinsing of *Hypogymnia* thalli led to a substantial signal increase in this chlorophyll fluorescence area in all inspected samples.

### 3.4. Fluorescent Secondary Substance Distribution Within Lichen Thalli

Cross-sections of *Hypogymnia physodes* and *H. bitteri* thalli were studied using laser scanning confocal microscopy to visualize fluorescent secondary lichen metabolite deposition. The yellow signal attributed to atranorin and chloroatranorin fluorescence was detected across the whole thalli section; however, its distribution was highly uneven. The highest level of atranorin fluorescence belonged to the upper cortical layer, the thickness of which was 29.1 ± 2.5 μm (average ± SD) in *H. physodes* and 35.1 ± 2.7 μm in *H. bitteri* (Figure 3e). Within the cortex, atranorin was deposited mainly on the thallus surface in *H. physodes* (Figure 3e) and in *H. bitteri* (Figure S1a,b). In algal layer (44.1 ± 7.5 μm in both species), atranorin was deposited on the surface of some hyphae and around algae cells. In medullary layer (104.4 ± 8.3 μm and 132.4 ± 10.5 μm, respectively), atranorin distribution was scarce; however, it was accumulated in a number of bright spots, which in some cases were located on hyphae tips. Lower cortical layer was slightly thicker and heavily melanized in both *H. physodes* (35.3 ± 5.6 μm) and *H. bitteri* (43.0 ± 4.5 μm). Importantly, atranorin and chloroatranorin were detected there as well (Figure S1e). However, the fluorescence signal from the lower cortical layer never reached its level from the upper cortical layer, being slightly higher than from the medullary layer.

Acetone rinsing substantially decreased fluorescence signal from atranorin and chloroatranorin in upper cortex and algal layer of *H. physodes* (Figure 3f) and *H. bitteri* (Figure S1b). In *H. bitteri*, thallus aging was accompanied by melanization of the cortical layer, where atranorin and chloroatranorin were gradually replaced by melanin (Figure S1d). For this reason, the secondary metabolites exhibited an uneven distribution within the cortical layer of the thallus: some lichen substances were located in non-melanized areas, while the remainder occurred as a thin layer between the melanized parts of the cortex (Figure S1d). This unusual localization of atranorin and chloroatranorin in the lower cortex explains the absence or very weak fluorescence of the thallus surface of *H.* *bitteri* well, despite the content of these secondary metabolites being comparable to that of *H.* *physodes*.

### 3.5. Fluorescence Spectra of Individual Metabolites

**Figure 4:**
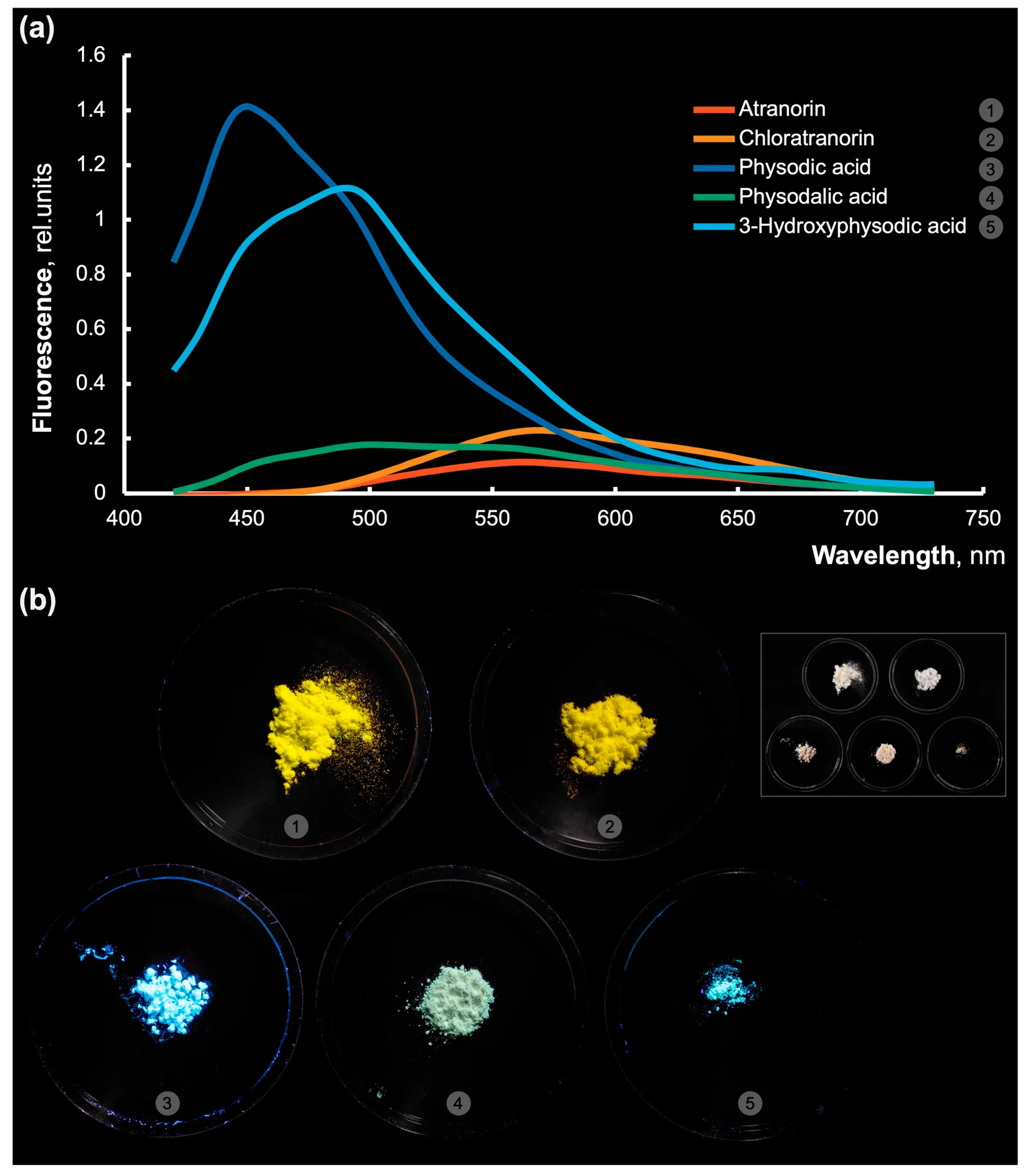
Fluorescence spectra curves (**a**) of the atranorin (red line, 1), chloroatranorin (orange line, 2), physodic acid (blue line, 3), physodalic acid (green line, 4) and 3-hydroxyphysodalic acid (cyan line, 5) excited at 365 nm UV light. Each curve represents the mean of five replicate measurements. (**b**) Pure dry substance fluorescence excited at 365 nm and photographed in visible light: atranorin (1), chloroatranorin (2), physodic acid (3), physodalic acid (4) and 3-hydroxyphysodic acid (5). Inset on the right: same pure substance specimens under visible light illumination.

Investigation of the fluorescence spectra of major individual metabolites found in the studied lichens showed that all these compounds exhibited fluorescent properties (Figure 4a). The identified emission maxima of atranorin, chloratranorin, physodalic acid, 3-hydroxyphysodic acid, and physodic acid were at 563, 566, 500, 490, and 450 nm, respectively (Figure 4a). The greatest fluorescence brightness was demonstrated by the medullary depsidones 3-hydroxyphysodic acid and physodic acid, with emission maxima in the blue–cyan part of the spectrum. Atranorin and chloroatranorin fluorescence maxima were in yellow part of the spectrum. These major secondary substances showed the observed fluorescence colors corresponding to their spectral properties: pure atranorin and chloroatranorin fluorescence was perceived as yellow and moderate yellow-orange, respectively (Figure 4b). The fluorescence of physodic and 3-hydroxyphysodalic acids appeared as cyan, and physodalic acid fluorescence was barely visible as a bluish tint over creamy white crystals.

### 3.6. PAM Fluorimetry Study of Hypogymnia Thalli

**Figure 5:**
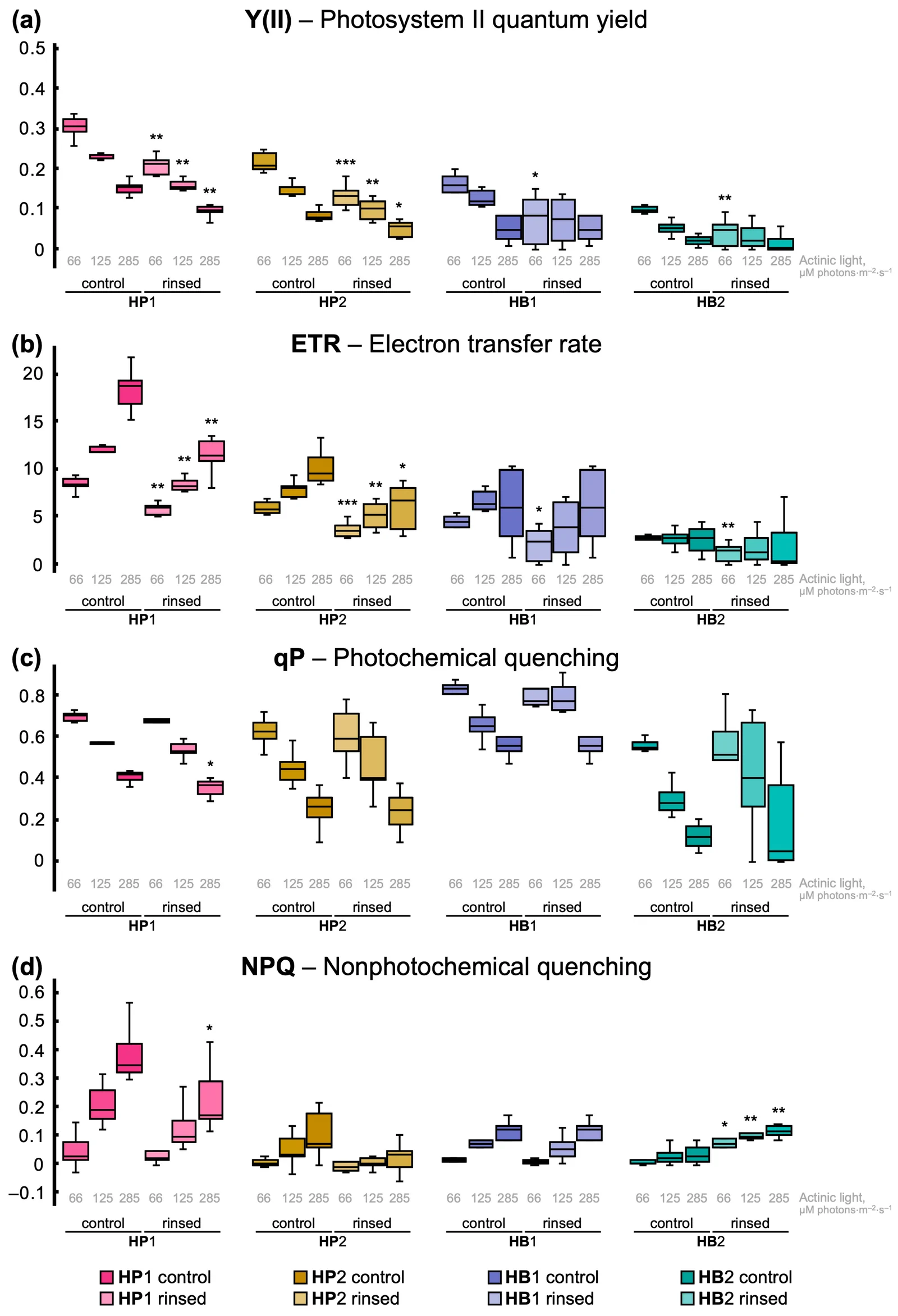
Pulse-amplitude modulation fluorescence (PAM) study of *Hypogymnia physodes* (HP1 and HP2) and *H. bitteri* (HB1 and HB2) thalli before (control) and after acetone rinsing. Lichens were illuminated with progressively increasing actinic light of three intensity levels from low to moderate: 66, 125 and 285 μM photons·m^−2^·s^−1^. (**a**) Photosystem II quantum yield (Y(II)), dimensionless values. (**b**) Electron transfer rate (ETR) through electron transport chain of chloroplast, μmol electrons·m^−2^·s^−1^. (**c**) Photochemical quenching (qP), dimensionless values. (**d**) Non-photochemical quenching (NPQ), dimensionless values. Asterisk marks the groups whose values are significantly different from corresponding control ones (*n* = 5, *—*p* < 0.05, **—*p* < 0.01, ***—*p* < 0.001, Mann–Whitney test).

Pulse-amplitude modulation (PAM) fluorimetry allows for non-destructive study of photosynthetic apparatus conditions in plants, algae and cyanobacteria [18]. In this research, PAM fluorimetry was employed to characterize the response of *Hypogymnia* photobiont (*Trebouxia* species) [19,20] to various illumination levels and to the depletion of atranorin and chloroatranorin in thallus resulting from acetone rinsing. Intact (control) thalli of both *Hypogymnia physodes* and *H. bitteri* showed significantly higher photosystem II quantum yield (Y(II)) (0.22–0.31 and 0.10–0.15, respectively) than acetone-rinsed ones (0.13–0.21 and 0.04–0.07, respectively) under low actinic light (Figure 5a). Progressively increasing actinic light caused a decrease in Y(II) in intact thalli of both species, as expected. However, acetone-rinsed *H. bitteri* demonstrated equally low Y(II) under low and moderate actinic light. The electron transfer rate (ETR) in the chloroplast electron-transfer chain naturally increased with actinic light intensity in control thalli of *H. physodes* from 6.03–8.46 to 9.63–18.36 and in *H. bitteri* from 2.66–4.07 to 2.73–9.38 μmol electrons·m^−2^·s^−1^ (Figure 5b). Acetone-rinsed *H. physodes* thalli showed significantly lower ETR (increasing from 3.76–5.77 to 1.13–11.53 μmol electrons·m^−2^·s^−1^) than control ones. In contrast, ETR in acetone-rinsed *H. bitteri* thalli was significantly lower than in control ones only under dim actinic light (1.16–2.09 μmol electrons·m^−2^·s^−1^).

Photochemical quenching (qP) is the fraction of harvested light energy utilized in photochemical processes of light reactions and the subsequent carbon reactions of photosynthesis. The highest qP values were demonstrated by control thalli of *H. bitteri* (0.81) and *H. physodes* (0.70) under low light (Figure 5c). Increasing actinic light intensity decreased qP in control as well as in acetone-rinsed specimens, mostly without significant differences between treatment groups. Non-photochemical quenching (NPQ) is induced in photosynthesizing organisms under excessive light and occurs thanks to heat energy dissipation from xanthophylls. In our study, NPQ rapidly increased in lichen thalli with increasing actinic light intensity (Figure 5d). In *H. physodes* HP1 and HP2, NPQ under moderate actinic light was slightly lower in control thalli than in acetone-rinsed ones. In HB1, NPQ was the same in control and rinsed thalli. Remarkably, acetone-rinsed HB2 thalli demonstrated 4.1–5.8 times higher NPQ than control thalli (Figure 5d). This response perfectly aligns with the data on the lowest remaining amount of atranorin and chloroatranorin in HB2 thalli after acetone rinsing (Section 3.1; Figure 2).

## 4. Discussion

### 4.1. Composition and Localization of Secondary Metabolites in Hypogymnia

The present study revealed that samples of *Hypogymnia physodes* (HP1 and HP2) contained five major secondary metabolites (physodalic acid, 3-hydroxyphysodic acid, physodic acid, atranorin, and chloroatranorin), whereas samples of *H. bitteri* (HB1 and HB2) contained six major secondary metabolites (vittatolic acid, 2′-O-methylphysodic acid, α-alectoronic acid, physodic acid, atranorin and chloroatranorin), which belong to depsides and depsidones (Figure 1, Table 1). The compositional profiles of secondary metabolites in *H. physodes* and *H. bitteri* were consistent with those previously reported in the literature [21,22].

HPLC analysis showed that acetone rinsing of air-dried *H. physodes* and *H. bitteri* lichens did not result in complete removal of secondary metabolites from the thalli (Figure 1). Atranorin and chloroatranorin, localized in the cortical layer, were less effectively rinsed by acetone compared to depsidones located in the medulla (Figure 3e,f and Figure S1a,b). Previous studies have also reported the inability to fully remove usnic acid, vulpinic acid, and atranorin from the lichen cortical layer following acetone rinsing [9,10,11]. This observation is likely attributable to the structure of the lichen cortical layer. In the dry state, this layer is characterized by densely packed hyphal cells, with interhyphal spaces filled by a viscous, hydrophilic extracellular matrix composed of β-glucan polysaccharides [23,24]. It can be assumed that acetone treatment results in the removal of crystals of depsidones located on the hyphal surface in medulla, whereas atranorin and chloroatranorin crystals embedded within the deeper, denser layers of the cortical layer remain inaccessible to acetone rinsing. Presumably, on the one hand, the denser packing of atranorin and chloroatranorin crystals within the extracellular matrix may provide more effective photoprotection. On the other hand, such atranorin and chloroatranorin localization may modulate interactions between the mycobiont and other surface-associated microorganisms owing to the bactericidal and fungicidal activity of these compounds [25]. The inability to completely remove lichen substances from the cortical layer is a limiting factor in studies of the ecological role of lichen metabolites. Overcoming this limitation apparently requires the selection of different solvents or methodological approaches.

### 4.2. Atranorin and Chloroatranorin Fluorescence and Its Contribution to Lichen Thallus Reflectance

Previous studies have investigated the capacity of lichen cortical compounds to act as photoprotective screens by reflecting light across the visible spectrum [10,12,26]. The surface reflectance of air-dried *H. physodes* and *H. bitteri* lichens was investigated in this study (Figure 3a). Comparison of reflectance spectra showed that acetone rinsing of *H. physodes* and *H. bitteri* thalli led to slight reduction in thallus surface reflectance across all studied samples compared to the acetone-untreated controls. The pale *H. physodes* (HP1 and HP2), exhibited significantly higher reflectance compared to the partially melanized *H. bitteri* (HB1 and HB2), both before and after acetone rinsing (Figure 3a,c). Given that the reflectance maximum within the visible range of the spectrum was at 560 nm (Figure 3a), thalli fluorescence maximum was near 560 nm as well (Figure 3b), pure atranorin and chloroatranorin UV-induced fluorescence maxima were in the same range (Figure 4a), and notable amount of these secondary substances were deposited in the cortical layer (Figure 3e), it should be concluded that atranorin and chloroatranorin themselves contribute to overall *H. physodes* thalli reflectance by fluorescence, since reflectance-measurement illuminating light contains short wavelengths. In contrast to *H. physodes*, darker *H. bitteri* thalli demonstrated substantially lower reflectance with no notable maxima in the visible range of the spectrum. Earlier studies revealed a considerable reduction in the reflectance of dark-brown lichen surfaces [27,28,29]. This is likely attributed to the accumulation of melanin in the cortex, which absorbs a portion of the visible spectrum incident on the lichen surface, thereby reducing its reflection. The thallus of *H. bitteri* not only contained a smaller amount of atranorin and chloroatranorin, but these substances were also likely replaced by melanin in the older parts of the thallus (Figure S1d).

The fluorescence of lichens in UV light was first demonstrated by Černohorský [30] with species of the genus *Parmelia*. Attention was drawn to the fluorescent properties of individual lichen substances quite a long time ago as well [13,15]. Currently, fluorescent properties are used for the identification of certain lichen substances in routine analysis by thin-layer chromatography, with plates irradiated by UV-A [14]. It has also been proposed that the fluorescent properties of lichen substances be used for their visualization and for studying their distribution patterns within the thallus by means of fluorescence microscopy [31,32].

It should be noted that a number of studies investigating the fluorescence of secondary lichen metabolites have also reported fluorescence data for substances dissolved in organic solvents [16,33]. These data are undoubtedly useful for more accurate identification of compounds during analysis, but they are not suitable for interpreting the physiological significance of the spectra obtained, because the fluorescence spectra of lichen substances in the form of crystals (the principal phase state in which most secondary metabolites occur in the lichen) differ considerably from those of the solutions. The present study has shown that orcinol depsidones in crystal state, located in the medullary layer, emitted intensely in the blue and blue-green regions of the spectrum (Figure 4), which is consistent with data reported previously [13,14]. At the same time, for most β-orcinol depsides and depsidones, fluorescence had previously been observed only in the blue part of the spectrum [13,14,15]. However, we have established that crystals of atranorin, chloroatranorin, and physodalic acid had fluorescence emission maxima in the yellow–green and green regions of the spectrum. Thus, colorless β-orcinol depsides and depsidones are capable of absorbing UV-A and blue–violet wavelengths and emitting fluorescence in the photosynthetically safe (unusable) yellow–green range of visible spectrum where neither chlorophylls nor carotenoids absorb light. This is especially important in the case of atranorin and chloroatranorin, given their localization in the cortical layer of the thallus. In addition to atranorin and chloroatranorin, *Hypogymnia* thalli contained compounds such as physodic acid that emit fluorescence in the blue range of the spectrum and are potentially able to transfer energy to chlorophylls. A similar relationship between parietin and chlorophyll in *Xanthoria* was already discussed in previous studies [16]. However, the intensity of light that reaches these medullary compounds in *Hypogymnia* is already substantially reduced due to the screening effect of atranorin and chloroatranorin in the cortical layer. Furthermore, physodic acid fluorescence induced by the remaining short-wavelength light is omnidirectional, so even less energy could be transferred to chlorophyll in algae. Therefore, physodic acid, 3-hydroxyphysodic acid and physodalic acid are unlikely to make a substantial contribution to photosynthesis in lichen thalli. We detected fluorescence from the upper surface of the thalli of *H. physodes* and *H. bitteri* upon excitation by wavelengths of 330–415 nm (Figure 3d). The fluorescence maximum of *H. physodes* was at 563 nm, which coincided with the emission maxima of atranorin and chloroatranorin (Figure 4a). As noted above, acetone rinsing did not completely remove atranorin and chloroatranorin from the cortical layer; consequently, only a partial decrease in the lichen’s fluorescence was observed. Previously, in a study by Kauppi and Verseghy-Patay [31], the yellow–green fluorescence of the cortical layer of *H physodes* under excitation by UV-A and blue–violet wavelengths had already been linked to the atranorin it contained, but the possible physiological significance of this effect was not considered. The most intense fluorescence was detected in specimen HP1, which was also characterized by the highest content of atranorin and chloroatranorin (Figure 2 and Figure 3b). At the same time, in *H. bitteri*, either no pronounced fluorescence peaks were observed (HB2) or fluorescence was very weak (HB1), both before and after acetone rinsing (Figure 3b).

### 4.3. A Possible Mechanism for the Photoprotective Role of Atranorin and Chloroatranorin

The key conclusion of the previous paragraph was that atranorin and chloroatranorin absorb UV-A and short wavelength range of the visible spectrum, and then emit this energy by fluorescence in yellow–green part of the spectrum, which is safe for *Trebouxia* photobiont [34,35,36]. Therefore, if these β-orcinol depsides play any photoprotective role in vivo, their removal from thalli is expected to impact algal photosynthesis, at least under high light intensities, and/or potentially decrease photobiont survival rate while thalli are dry. First, it is necessary to admit that acetone rinsing procedure itself affects photosynthesis yet is insufficient to completely remove atranorin and chloroatranorin from *Hypogymnia* thalli. Chlorophyll fluorescence measurements showed that photosystem II quantum yield and electron transfer rate decreased both in *H. physodes* and *H. bitteri* rinsed samples (Figure 5a,b; Section 3.6). Moderate or high light intensities induce non-photochemical quenching in the photobiont. Remarkably, the rise in NPQ was the highest in HB2 (Figure 5d), the thalli of which the highest amount of atranorin and chloroatranorin was rinsed off. It is well-established that multiple secondary compounds participate in light screening of the lichen photobiont [10,11,12,26,29,37,38,39]. Compounds such as usnic acid and isousnic acid absorb high-energy photons of UV-A, UV-B and blue–violet light and dissipate that energy as heat, potentially interfering with photosynthesis carbon reactions and/or affecting the stability of photosystems and antennae proteins. On the other hand, these substances contribute to overall albedo of the lichen thallus when located in the cortical layer. Removal of secondary metabolites capable of screening short-wavelength light increases photodamage during desiccation and forces the photobiont to increase NPQ. In contrast, melanization of thalli acts in a less specific manner by light absorption in significantly wider range of spectrum. Therefore, melanized lichens such as *Cetraria islandica* demonstrate generally lower NPQ and increase NPQ slower than usnic lichens (*Cladonia stellaris*) when exposed to high light [29]. In our study, we compared non-melanizing *H. physodes* with *H. bitteri*. Given that the latter species is capable of strong melanization in mature parts of the thallus, we analyzed chlorophyll fluorescence exclusively in the young part of thalli having the least possible melanization. This part of thalli naturally responded to atranorin and chloroatranorin depletion with NPQ increase under increasing light intensity. Remarkably, dry atranorin and chloroatranorin are capable of not only absorbing and dissipating UV-B, reflecting and scattering the descending light, but also re-emit the absorbed short-wavelength component as omnidirectional yellow–green fluorescence in a temperature-neutral and photosynthetically safe manner.

## 5. Conclusions

Thus, we identified another protective mechanism of cortical β-orcinol depsides against excessive UV-A and short-wavelength visible radiation, which involves absorption and re-emission in the photosynthetically safe yellow–green part of the spectrum. Together with other known photoprotective properties of secondary lichen metabolites and melanins, this mechanism ensures their protection and stable survival under harsh growing conditions. In addition, special attention should be paid to developing new methods for removing secondary metabolites from the thallus using milder solvents instead of acetone. Such solvents should both extract lichen substances from the thallus more efficiently and cause less damage to the photobiont. Furthermore, more detailed studies with larger sample sizes are needed to investigate the effect of sunlight intensity on the accumulation of protective metabolites in lichens, taking into account the photoprotective properties of atranorin and chloroatranorin identified in the present study.

## Figures and Tables

**Table 1:** The lichen substances in *Hypogymnia physodes* and *H. bitteri* thalli identified by HPLC-ESI-MS/MS.

Peak No.	Rt (min)	Molecular Formula	[M − H]^−^ *m*/*z*	MS/MS Fragments	Identification	Type *
1	12.1	C_20_H_16_O_10_	415	355, 311, 296, 283, 267	Physodalic acid	β-OD
2	12.7	C_26_H_30_O_9_	485	441, 423, 399, 379, 369	Vittatolic acid	OD
3	14.4	C_26_H_30_O_9_	485	441, 423, 399, 397, 373	3-Hydroxyphysodic acid	OD
4	16.4	C_27_H_32_O_8_	483	439, 424, 407,395, 383	2′-O-methylphysodic acid	OD
5	16.9	C_26_H_30_O_8_	469	425, 407, 383, 381, 357	Physodic acid	OD
6	18.1	C_28_H_32_O_9_	511	467, 449, 425, 423, 369	α-alectoronic acid	OD
7	21.5	C_19_H_18_O_8_	373	177, 163, 133, 119, 105	Atranorin	β-Od
8	23.0	C_19_H_17_ClO_8_	407	211, 167, 163, 139, 119	Chloroatranorin	β-Od

* β-Od = β-orcinol depside; β-OD = β-orcinol depsidone; OD = orcinol depsidone [5].

## Data Availability

The data presented in this study are available on request from the corresponding author. The data are not publicly available due to privacy restrictions.

## Supplementary Materials

The following supporting information can be downloaded at https://www.mdpi.com/article/10.3390/jof12100768/s1, Figure S1: Features of atranorin and chloroatranorin localization in *Hypogymnia bitteri* and *H. physodes* thalli.

## Abbreviations

The following abbreviations are used in this manuscript:

ETR	Electron transfer rate through electron transport chain of chloroplast
HB1, HB2	*Hypogymnia bitteri* specimens from location 1 and 2, respectively
HP1, HP2	*Hypogymnia physodes* specimens from location 1 and 2, respectively
NPQ	Non-photochemical quenching
PAM	Pulse-amplitude modulation (fluorimetry)
qP	Photochemical quenching
Y(II)	Photosystem II quantum yield
