# Supplementary material for: Atranorin Fluorescence: A Possible Photoprotective Role in Lichen Defense Against UV and Blue–Violet Radiation
Source: J Fungi (Basel). 2026 Oct 13;12(10):768. doi: 10.3390/jof12100768 (PMC13705759; doi:10.3390/jof12100768)
Supplement: Supplementary file 1 [file jof-12-00768-s001.zip › Figure S1.pdf]

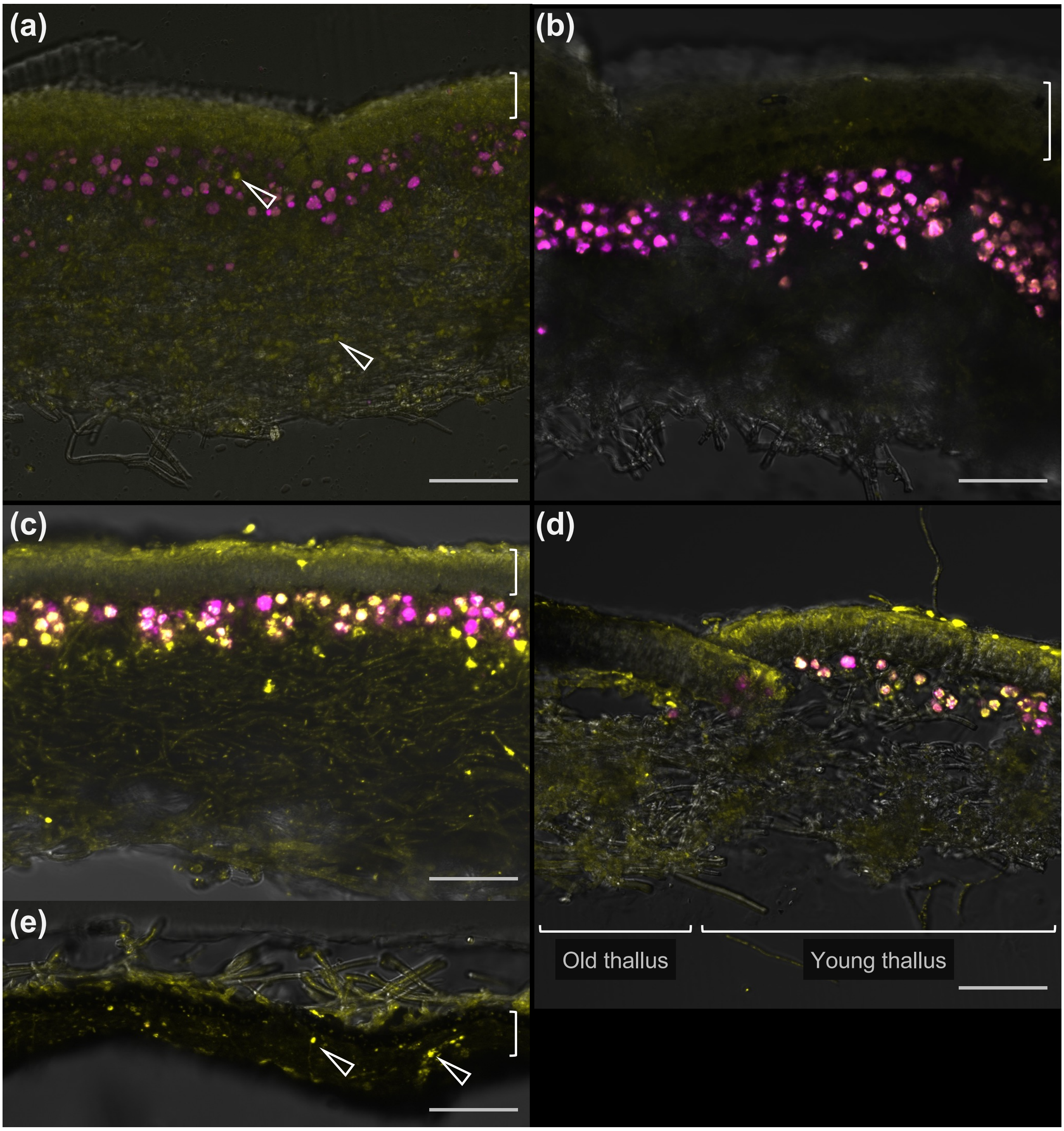

Figure S1. Features of atranorin and chloroatranorin localization in *Hypogymnia bitteri* and *H. physodes* thalli. **(a)** Cross-section of *Hypogymnia bitteri* (HB1) young thallus before acetone rinsing (control). **(b)** HB1 thallus cross-section after acetone rinsing. Upper cortical layer is significantly depleted of atranorin and chloroatranorin fluorescence (marked with bracket). **(c, e)** Cross-section of control *Hypogymnia physodes* upper part **(c)** and lower **(e)** cortical layer. **(d)** Transition from young control *Hypogymnia bitteri* thallus accumulating atranorin in the upper cortex (right) to the older thallus (left) that is highly melanized. Images represent maximal intensity projections from 8–14 confocal optical sections. Atranorin and chloroatranorin fluorescence (yellow) and chlorophyll autofluorescence (magenta) were excited by 405 nm diode laser. Monochrome background: transmission channel rendered by 594 nm laser. Arrows indicate fluorescent secondary metabolites accumulated in spots in cortex, algal or medullary layers. Brackets indicate thallus cortex. Scale bars: 50 μm.
